# Cluster Randomized Controlled Trial on the Effects of 12 Months of Combined Exercise Training during Hemodialysis in Patients with Chronic Kidney Disease—Study Protocol of the Dialysis Training Therapy (DiaTT) Trial

**DOI:** 10.3390/mps4030060

**Published:** 2021-08-31

**Authors:** Gero von Gersdorff, Pia von Korn, André Duvinage, Gabriele Ihorst, Anika Josef, Margit Kaufmann, Thomas Baer, Tim Fellerhoff, Iris Fuhrmann, Elisa Koesel, Sven Zeissler, Lars Bobka, Marion Heinrich, Anette Schindler, Rasmus Weber, Cornelia Breuer, Anna Maria Meyer, M. Cristina Polidori, Sophia M.T. Dinges, Julia Schoenfeld, Mathias Siebenbuerger, Stefan Degenhardt, Kirsten Anding-Rost, Martin Halle

**Affiliations:** 1 QiN-Group, Department II of Internal Medicine, Faculty of Medicine and University Hospital Cologne, University of Cologne, 50935 Cologne, Germany; gero.freiherr-von-gersdorff@uk-koeln.de (G.v.G.); cornelia.breuer@uk-koeln.de (C.B.); 2 Department of Prevention and Sports Medicine, Faculty of Medicine, University Hospital ‘Klinikum rechts der Isar’, Technical University Munich, 80992 Munich, Germany; Pia.vonKorn@mri.tum.de (P.v.K.); Andre.Duvinage@gmx.de (A.D.); baer.thomas.1977@gmail.com (T.B.); tim.fellerhoff@gmail.com (T.F.); iris-fuhrmann@arcor.de (I.F.); Elisa.Koesel@mri.tum.de (E.K.); svenzeissler@gmx.net (S.Z.); Sophia.Dinges@mri.tum.de (S.M.T.D.); Julia.Schoenfeld2@mri.tum.de (J.S.); mathias.siebenbuerger@gmx.de (M.S.); Kirsten.Anding-Rost@kfh.de (K.A.-R.); 3 DZHK (Deutsches Zentrum für Herz-Kreislauf-Forschung), Partner Site Munich, Munich Heart Alliance, 80336 Munich, Germany; 4 Clinical Trials Unit, Faculty of Medicine and Medical Center, University of Freiburg, 79110 Freiburg, Germany; gabriele.ihorst@uniklinik-freiburg.de (G.I.); anika.josef@uniklinik-freiburg.de (A.J.); margit.kaufmann@uniklinik-freiburg.de (M.K.); 5 Kuratorium für Dialyse und Nierentransplantation e.V. (KfH), 01877 Bischofswerda, Germany; 6 Deutsche Gesellschaft Rehabilitationssport für chronisch Nierenkranke e.V. (ReNi, Germany), 01445 Bischofswerda, Germany; stefan.degenhardt@web.de; 7 BARMER, 01069 Dresden, Germany; lars.bobka@barmer.de (L.B.); marion.heinrich@barmer.de (M.H.); 8 AOK PLUS, 01067 Dresden, Germany; anette.schindler@plus.aok.de; 9 Techniker Krankenkasse, 22305 Hamburg, Germany; Rasmus.Weber@tk.de; 10 Ageing Clinical Research, Department II of Internal Medicine, Faculty of Medicine and University Hospital Cologne, University of Cologne, 50931 Cologne, Germany; anna.meyer@uk-koeln.de (A.M.M.); maria.polidori-nelles@uk-koeln.de (M.C.P.); 11 Center for Molecular Medicine Cologne, Faculty of Medicine and University Hospital Cologne, University of Cologne, 50931 Cologne, Germany; 12 Cologne Excellence Cluster on Cellular Stress-Responses in Aging-Associated Diseases (CECAD), Faculty of Medicine and University Hospital Cologne, University of Cologne, 50931 Cologne, Germany

**Keywords:** exercise, frailty, quality of life, health economics, kidney failure, end stage renal disease

## Abstract

Patients with chronic kidney disease (CKD) on hemodialysis (HD) experience treatment-related immobility and physical deconditioning, which is responsible for an increased risk of frailty and a high burden of multi-morbidity. Exercise has been shown to counteract this vicious cycle; however, its effectiveness has only been investigated in small cohorts. Therefore, the objective of the Dialysis Training Therapy (DiaTT) trial will be to assess the effects of a 12-month intradialytic exercise program on physical functioning, frailty and health economics in a large cohort of HD patients in a real-world setting. DiaTT will be a prospective, cluster-randomized (1:1), controlled, multi-center, interventional clinical trial across 28 dialysis units, aiming at the recruitment of >1100 CKD patients on HD. The intervention group will receive 12 months’ intradialytic exercise (combined aerobic and resistance training), whereas the usual care group will not receive intervention. The primary endpoint will be a change on the sit-to-stand test (STS60) result between baseline and 12 months. Secondary endpoints will include physical functioning, frailty, quality of life, 3-point MACE, hospitalizations, survival, quality of HD, health literacy and health care costs. By including almost as many patients as previously investigated in smaller trials, DiaTT will be the largest randomized, controlled trial assessing frailty, quality of life and mortality in the field of nephrology.

## 1. Introduction

Many patients with chronic kidney disease (CKD) suffer from marked deconditioning, leading to a vicious cycle of inactivity and a further decline in physical functioning [1]. The prevalence of frailty in patients with CKD ranges from 7% in community-dwellers (CKD Stages 1–4) to 73% in cohorts of patients treated with hemodialysis (HD) and has a direct impact on increased morbidity and mortality [2]. This was also observed in the Dialysis Outcomes and Practice Patterns Study (DOPPS, n = 7226, mean age 63.6 ± 14.4 years, median follow-up 17.2 months, self-reported functional status) [3], which showed that a very high proportion of dialysis patients, independent of age, experience difficulties in the performance of routine daily tasks and that low physical functioning was strongly associated with low quality of life and increased mortality [3,4].

Exercise training has been shown to counteract the decline of physical functioning in CKD. In a recent meta-analysis of fifty studies including n = 1757 CKD patients on HD, aerobic intradialytic training improved peak exercise capacity (VO_2_peak) by 2.07 mL/kg/min and 6-min walk test distance by 65 m [5]. Adding resistance to aerobic endurance training was even more effective, increasing VO_2_peak by 5.41 mL/kg/min [5]. Similar findings were reported in a meta-analysis of 27 studies including 1156 participants, showing that exercise programs, regardless of modality, generally increased physical performance parameters, such as sit-to-stand time or repetitions [6]. However, up to now, randomized trials of exercise interventions during HD have only included smaller cohorts, e.g., up to n = 228 patients [7], and the majority have tested interventions only for a short duration, e.g., 12 weeks [8]. Only very few studies evaluated longer intervention periods up to 9 months [7]. Moreover, a number of barriers to the implementation of exercise routines have been identified [1]. These include the widespread skepticism of dialysis staff towards exercise programs during dialysis, questions surrounding the suitability of very sick patients for exercise and the fear that exercise programs may interfere with the carefully orchestrated care processes of dialysis units and cause additional workload. In addition, patients treated with dialysis report a number of barriers to exercise, including hospitalizations, pain, fatigue and others.

However, contrary to this skepticism, in our experience of routine practice as well as our pilot study (46 HDpatients, age 63.2 ± 16.3 years, observation period up to 5 years), a supervised intradialytic exercise program, combining resistance and aerobic training twice weekly for 60 min, significantly improved exercise capacity, strength, quality of life and physical functioning and was associated with a high adherence rate of 78% after 1 year and 43% after 5 years, without safety issues [9]. Exercise was conducted by exercise therapists and did not require the involvement of the regular dialysis staff; it allowed the participation of patients with almost all clinical conditions or levels of disability and was found to integrate well with care processes in the dialysis unit.

The same concept will be applied in the current DiaTT trial, in which we will extend this experience from a single center to a multi-center context and substantially expand patient numbers, applying as few medical restrictions on participation as possible. By including only patients from one non-profit dialysis provider, the established dialysis structure, clinical assessment, clinical chemistry and data handling will be identical for all sites. The primary objective will be to assess the effectiveness of exercise training during HD compared to usual care (UC; no intervention) after 12 months. The intervention will combine strength and aerobic exercise training and add health literacy counselling (HLC). We will investigate clinical outcomes, such as frailty, morbidity, mortality and health economics, in order to facilitate the future implementation of intradialytic exercise into routine health care reimbursement, a request that has been made repeatedly by the nephrological community [6,10,11,12]. 

By including >1100 well-documented CKD patients in this large randomized study, the DiaTT trial, after completion, will be one of the three largest randomized, controlled trials comparing exercise intervention with the usual care in medicine, together with the HF ACTION trial, which included 2331 chronic heart failure patients over 30 months [13] and the Look AHEAD trial, which included 5145 obese patients with type 2 diabetes over 9.6 years [14]. 

## 2. Experimental Design and Methods

### 2.1. Study Design

**Figure 1:**
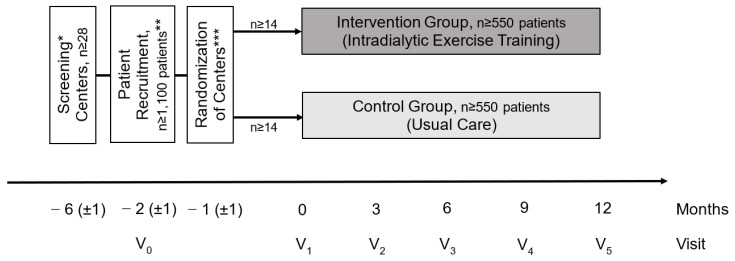
Study design of the DiaTT trial, including study visits (V). * From all the KfH dialysis centers in five regions in Germany, 28 will be recruited. ** In these centers, patients will be screened for eligibility (inclusion/exclusion criteria, informed consent) and included in the study (V0 screening). *** Centers will be randomized (1:1) to Intervention Group and Control Group. Visits will be at baseline (V1) and after 3, 6, 9 and 12 months (V2–V5).

DiaTT will be an interventional, cluster randomized (1:1), multicenter, controlled trial. After screening of dialysis centers and patient recruitment, 28 centers will be cluster randomized to IG or UC, as depicted in Figure 1. The randomization of centers will be stopped once a minimum of n = 1100 patients have been recruited. The intervention will consist of 12 months’ combined exercise training (aerobic and resistance), initiated after the baseline visit (V1), compared to UC without intervention.

The primary endpoint will be a change in the Sit-to-Stand (STS60) test result [15] from baseline to 12 months between groups. Secondary endpoints will include physical functioning (Timed-Up-and-Go test (TUG)), the Six-Minute Walk test (6MWT), the Grip Strength Test (GST)), frailty (Multidimensional Prognostic Index (MPI)), quality of life (SF-36), 3-point Major Adverse Cardiovascular Events (MACE), hospitalizations, overall survival, quality of HD (measured as serum phosphate and other laboratory values), medication (phosphate binders, erythropoietin), health literacy (HLS-EU-Q16 Health Literacy Questionnaire) and health economics (total costs of medication, hospitalization, therapeutic remedies, transportation, medical assistance tools, nursing care and sick leave from workplace). 

The study has been registered at ClinicalTrials.gov NCT03885102.

### 2.2. Center Eligibility 

The trial will be conducted in up to 28 dialysis centers of the non-profit kidney care provider KfH in Germany (Kuratorium für Dialyse und Nierentransplantation e.V.; Board of Trustees for Dialysis and Kidney Transplantation). Centers in the federal states of Saxony-Anhalt, Thuringia, North-Rhine Westphalia and Bavaria will be informed and, if interested, screened according to the following inclusion criteria: willingness to accommodate the training program in their center, adequate space for training and the storage of equipment and a minimum of n = 50 HD patients. Centers with an existing exercise program during HD will be excluded from participation.

### 2.3. Cluster Randomization of Centers

Participating centers will be randomly assigned to IG or UC using stratified block randomization with a block size of two centers and stratified by region. To ensure concealment, two centers from the same stratum will be randomized at the same time. 

### 2.4. Study Population

All patients from the participating dialysis centers will be asked to participate in the trial during the screening visit (V0). 

The inclusion criteria for CKD patients will be: age ≥ 18 years, chronic ambulatory HD for >4 weeks, written and signed confirmation by the treating dialysis physician that the patient is able to exercise, signed informed consent to participation on the Quality-in-Nephrology (QiN) registry, signed informed consent obtained according to confidentiality and data protection regulations, international guidelines and local laws. 

The exclusion criteria will be: unstable angina pectoris, uncontrolled arterial hypertension (systolic blood pressure > 180 mmHg or diastolic BP > 105 mmHg on repeated measurements), uncontrolled tachycardia, acute severe infection, planned live kidney transplantation within the next 12 months, planned conversion to home-HD or peritoneal dialysis within the next 12 months, long overnight dialysis, participation in a regular exercise program during HD sessions (≥1/week) in the last 6 months. 

Irrespective of the randomized center group assignment, all dialysis and other medical treatment procedures will follow standards of care and center routine without adaptations to the training intervention. Patients of both sexes will be enrolled and no study-specific sex distribution is foreseen. Patients who are hesitant about exercising because of their medical conditions or disabilities will be reassured and encouraged to participate, as long as their condition does not meet the exclusion criteria.

## 3. Procedure

### 3.1. Intervention and Usual Care Groups

#### 3.1.1. Usual Care 

Patients will receive general information on the importance of physical activity, nutrition and exercise opportunities, which are available to patients on dialysis under current health insurance laws and regulations. Routine dialysis care will continue according to local standards and practices without modification. 

#### 3.1.2. Intervention Group (IG)

In the IG, patients will participate in a standardized and individually adapted intradialytic exercise training program, supervised by specially trained exercise therapists. Patients will additionally receive HLC by the exercise therapists while exercising. The exercise intensity will be increased monthly according to standard operating procedures and reassessed every three months. Exercise therapists will continuously promote adherence and motivation to exercise intervention. 

The intervention will consist of a combined exercise program (aerobic and resistance training) thrice-weekly during HD. Each exercise session will last 60 min, with a short break between exercise modes (30 min cycling and 30 min of resistance training). The exercise training will be performed within the first two hours of the HD session. 

##### Aerobic Training

All aerobic exercises and tests will be performed on a bed-cycle ergometer in a semi-recumbent position, which adapts workload to the continuously monitored target heart rate (MOTOmed letto2, Reck-Technik GmbH & Co KG Medizintechnik, Betzenweiler, Germany) and has been previously used in patients with CKD for intradialytic training [9]. If needed, an electric support while pedaling can be programmed to engage weaker patients in cycling. 

The target heart rate (HR) will be determined by the Karvonen formula [HR_Training_ = (HR_max_ − HR_rest_) × Factor + HR_rest_] at baseline and on the six-month visit. Endurance training will start with a load of 2, 6, or 12 watts (according to the patient’s physical status) and increase stepwise until voluntary or symptom-limited exhaustion. The training should be perceived as moderately strenuous (Borg Scale of perceived exertion: 12–13, “somewhat hard”). The training intensity will increase every four to eight weeks by recalculating the target heart rate using a factor of 0.5 for the first month and a factor of 0.75 at month 5. After six months of training, HR_Training_ will be reassessed and the intensity will again increase stepwise by increasing the target heart rate using factors from 0.65 to 0.75. Thus, individualized training parameters will be programmed on a patient´s ergometry-ID card to facilitate an adaptive response by the cycle ergometer based on HR during training. The training data recorded by the ergometer will be regularly uploaded to the study database (eCRF). 

##### Resistance Training

Resistance training will be comprised of eight exercises for upper and lower limbs performed with dumbbells and elastic bands of varying resistance, as well as core strength exercises, in a semi-recumbent position. Exercises will be modified in accordance with patients’ functional status and disabilities (e.g., limb amputation) and adapted from previous experience [9].

Strength tests will be performed every three months to determine the one-minute maximum repetition rate for each exercise. The training intensity will be derived from these tests to achieve a perceived exertion of 12–13 (“somewhat hard”) on the Borg scale and will increase each month by increasing the number of repetitions, weight or resistance, according to standardized protocols. Exercises will be performed in two sets of one minute each with a one-minute break. Biceps and triceps are trained with weights (dumbbells) of 0.5, 1, 2, 4 or 7 kg. For the abductors, elastic bands (Theraband^®^) with different resistances will be used; rubber balls of varying sizes will be used to support adductor and core strength exercises. If the number of repetitions exceeds 50 per minute, the next heaviest load will be applied. Strength training data will be recorded by exercise therapists on training logs at every session and entered into the study database (eCRF). 

In general, patients are advised to increase their daily physical activity on non-dialysis days, based on their individual physical functioning and caregiving level. Physical functioning levels are defined as low (no standing or walking possible), moderate (standing and guided walking is possible) or high (free standing and walking). Instructions for the exercises will be provided by their local exercise specialists. Patients will be advised to use common household items such as water bottles, cushions, chairs walls, benches and handrails.

##### Health Literacy Counseling

During or after the exercise sessions, the exercise therapists will give short topic presentations of five minutes each, covering nutrition/diet, fluid intake, physical activity, hypertension, diabetes, atherosclerosis, smoking, alcohol and stress. Topics will be rotated every few weeks and presented in lay language, structured around four topic headers (relevance, symptoms, treatment, pointers). They will be accompanied by leaflets with graphics, aiming to educate and empower patients regarding their disease and lifestyle choices.

Health literacy will be assessed in all patients at baseline and after twelve months using the European Health Literacy Questionnaire (HLS-EU-Q16) [16].

### 3.2. Visit Schedule

Patients in eligible centers will be informed (in writing and orally) and screened for eligibility at V0. Two dedicated members of the study staff will conduct the screening visits in all centers to ensure the consistent communication of study-related information and recruitment. After written informed consent is obtained, centers will be randomized to either IG or UC, respectively. Patients will undergo baseline examinations (V1) three months after screening (V0), after a roll-out phase for the delivery of exercise equipment and the recruitment of local exercise therapists at intervention sites. Clinical assessments and tests of physical functioning will be performed at V1 and repeated at 3, 6, 9 and 12 months (Visits 2–5). For details about the assessments see Table 1. 

To ensure consistency, only a dedicated group of specifically trained exercise therapists will conduct the assessments of physical functioning.

The documentation assistants in the dialysis units will receive specific training by geriatricians to administer MPI. Routine care parameters, which are recorded continuously in the digital patient registry (KfH-QiN), will be summarized automatically for each study visit and transferred to the eCRF.

Data on health economics will be derived from the participating patients who are insured at one of the three collaborating German health insurance companies (Techniker Krankenkasse, BARMER or AOK PLUS). 

### 3.3. Risk-Benefit Assessment

The risk of the exercise intervention for the patients in the current trial can be considered low, because of the close monitoring and supervision during HD by the dialysis staff and trainers. In our pilot study, no serious adverse events (SAEs) related to intradialytic exercise were noted [9], which was consistent with findings in previous trials from other groups [7]. 

### 3.4. Adverse Events and Serious Adverse Events 

The following adverse events (AE), potentially linked to intradialytic exercise, will be recorded throughout the study: venous needle displacement or catheter disconnection during HD symptomatic hypotension during HD (clinically symptomatic, requiring medical intervention) and episodes of cramping (calves) during HD (requiring medical intervention). 

The following serious adverse events (SAE) will be recorded on the eCRF regardless of their occurrence during or between HD sessions: hospitalization (admission or discharge diagnosis) and death, including date and cause of death.

### 3.5. Study Staff

A team of exercise coordinators, under the supervision of the coordinating investigator of the study, will perform screening visits, supervise and organize the implementation of the study in the roll-out phase (between V0 and V1), supervise and participate in all study visits, and monitor adherence to the training and SOPs by the local exercise therapists.

The local exercise teams (three to five trainers per dialysis center) will receive special training ahead of the intervention by experts in exercise nephrology (ReNi e.V., Berlin, Germany: Society for Exercise in the Rehabilitation of CKD Patients) and conduct every training session at IG center site.

In addition, one documentation assistant (from the dialysis nursing staff) at each study site will be responsible for coordinating the study visits and for the documentation of all the study data (not related to the exercise intervention) and physical functioning assessments. 

### 3.6. Data Handling and Data Management

Data will be managed with REDCap™ Version 8.6.5, a fully web-based remote electronic case report form (eCRF; REDCap Consortium, redcap@vanderbilt.edu). Data input into the primary eCRF database will be derived from several sources: 1. Direct input on the web-based eCRF, 2. Digitized survey data (questionnaires), 3. Uploaded training data from cycle ergometers and 4. Data extraction from the digital KfH-QiN dialysis registry via a custom interface. All patient data will be stored under a unique DiaTT identifier (DiaTT-ID) without other identifying information. 

The KfH-QiN patient registry is a digital registry database [17], which has been established before and independently of the DiaTT study for the quality improvement, analysis and scientific research of routine patient care data in all KfH dialysis centers. A specified dataset, which summarizes patient data collected between study visits, will be extracted from KfH-QiN and transferred into the DiaTT trial database. Data will be regularly reviewed for completeness, consistency and plausibility. There will be no on-site monitoring of data entry.

After the locking of the eCRF, the eCRF database will be merged with a claims dataset from participating health insurance companies, using the unique DiaTT-ID for matching.

This total DiaTT study database will be used for statistical analysis. Access to these data will be limited to the Clinical Trials Unit at Freiburg University for statistical analysis and to members of the steering board.

### 3.7. Statistical Planning and Analysis

Before the start of the final analysis, a detailed statistical analysis plan (SAP) will be prepared and completed during a “blind review” of the data before the database is locked. All statistical programming for analysis will be performed with the Statistical Analysis System (SAS Version 9.2, Cary, NC, USA).

#### 3.7.1. Sample Size Calculation

The sample size calculation is based on the primary objective of the trial, which is to demonstrate a difference between treatment groups after 12 months with respect to STS60. Assumptions are based on a single-arm trial in which the exercise training program was applied [9]. 

For STS60, mean ± standard deviation (SD) were reported: 16.7 ± 8.3 at baseline, 20.5 ± 8.8 after 6 months, 24.5 ± 10.2 after 12 months. Thus, a conservative assumption is 16.5 repetitions in the control group and 20.5 repetitions in the training group, i.e., a difference between treatment groups of 4 repetitions, and a standard deviation of 11.4. The corresponding standardized treatment effect (effect size) is then 4/11.4 = 0.35.

In order to detect a significant difference between the treatment groups at a two-sided level of α = 5% with a power of 80% under these assumptions, 130 patients per group (260 total) are necessary in an individually randomized trial. Cluster randomization must be considered in terms of a design factor. The average cluster size, m, is estimated as m = 50 patients. According to the CONSORT statement, small values (<0.05) are typical for the intraclass correlation coefficient (ICC). Thus, ICC = 0.05 represents a conservative assumption. The corresponding design factor is 1 + (m − 1) ICC = 3.45, and the required sample size is n = 897. Anticipating a mortality that is estimated to be <20%, at least 1100 patients will be randomized.

#### 3.7.2. Definition of Populations Included in the Analysis 

In the modified full analysis set (FAS), analyses of effectiveness will be performed according to the intention-to-treat (ITT) principle. All patients who have at least one post-baseline observation will be analysed in the treatment arms to which they were randomised, irrespective of whether they refused or discontinued the treatment or whether other protocol violations are revealed. A per-protocol (PP) analysis will be performed for sensitivity.

#### 3.7.3. Primary Endpoint 

The primary effectiveness analysis will be performed in the modified FAS. A linear mixed-effects regression model will be applied for the primary endpoint, STS60, at different time points, including randomized treatment, baseline STS60, the stratification factor region, time points, and a time*treatment interaction. Clusters will be included as a random effect, and repeated measurements of patients will be accounted for with an exchangeable correlation structure. The time*treatment interaction terms will enable the estimation of the therapy’s effect at different time points. The treatment effect estimates will be presented with two-sided 95% confidence intervals.

The primary analysis will be adjusted for baseline STS60 measurements, as proposed in the EMA ‘Guideline on adjustment for baseline covariates in clinical trials’ (EMA/CHMP/295050/2013, Section 5.6). 

A mixed model enables the incorporation of patients with partly incomplete observations. However, missingness at random (MAR) may be questionable. Sensitivity analyses will be performed with multiple imputation methods investigating the structure and impact of missing values.

#### 3.7.4. Secondary Endpoints for Effectiveness

The analysis of the secondary effectiveness endpoints will be performed with the same type of mixed regression model as described for the primary endpoint.

Overall survival (OS) is defined as the time from randomization until death from any cause, or as time from randomization until the patient was last alive (censored observations).

The OS rate will be estimated and described using the Kaplan-Meier method. Further analyses incorporating relevant prognostic factors will be conducted with Cox proportional hazards regression models.

Cause-specific survival will be defined as time from randomization until death from a specified cause (sudden death; 3-point MACE (a composite of cardiovascular death, non-fatal stroke, or non-fatal myocardial infarction)). Patients who are alive will be considered as censored observations and death from other causes will be considered as a competing event. The estimation of cumulative incidence rates will be applied in order to account for the presence of competing events. 

#### 3.7.5. Health Economics 

The health economic data will be analyzed descriptively and compared between the treatment groups using the FAS (i.e., medication (total costs), hospitalization (days in hospital, total costs), therapeutic remedies (total costs), transportation (total costs), medical assistance tools (total costs), nursing (total costs) and sick leave from the work-place (days)).

Additional health insurance data will be collected from patients insured with one of the participating health insurance companies. In order to assess whether the study population differs from typical German HD patients, basic characteristics will be compared, i.e., age, sex, nursing, comorbidities. 

## 4. Expected Results/Discussion

The DiaTT-trial will be the largest cluster randomized, controlled interventional trial for intradialytic exercise combining endurance and resistance exercise to date. It will address a need that is highly prevalent in many dialysis units and matters greatly to patients [18]. After the initiation of HD, many patients suffer from a pronounced, sustained decline in physical functioning, independent of sex, age, race or previous physical functioning (n = 3702 nursing home residents starting HD, activities of daily living +2.8 points, CI 2.5–3.0) [19]. The same is true for patients returning to ambulatory dialysis after prolonged hospitalizations. Without any treatment or intervention, 50% of CKD patients undergoing HD report a sedentary lifestyle, with exercise taken less than once a week [4] and a high proportion of frailty [3]. Frailty itself is associated with increased short-term (≤3 years, HR 2.18, 95% CI 1.76–2.70) and overall mortality (≥3 years, HR 1.71, 95% CI 1.20–2.44) in these CKD patients when compared to CKD patients without HD (meta-analysis including 12 cohort studies, n = 127037 patients with CKD, 2007–2019) [20]. Long hours without physical activity during HD, together with uremia-induced impaired neuromuscular functioning, low cardiorespiratory fitness and a high prevalence of disability and multi-morbidity [17] result in a progressive decline in physical functioning that is associated with low quality of life and high morbidity and mortality [1].This may be counteracted by active rehabilitation programs and exercise, as has been highlighted by several meta-analyses and reviews [3,5,6,21,22,23,24,25,26]. In particular, intradialytic exercise has been shown to improve physical functioning and cardio-pulmonary exercise capacity (peak oxygen consumption), as reported by Sheng et al. in 2014 (24 RCTs including n = 997 CKD patients on HD) [21] and Ferrari et al. in 2020 (50 RCTs; n = 1757 CKD patients on HD) [5]. 

### 4.1. Intervention Program

The optimal dose of intradialytic training has not been determined. However, intradialytic cycling alone (13 RCTs, n = 369 CKD patients on HD) shows equivocal results [27], whereas combinations of resistance training and aerobic exercise were found to be better at improving physical functioning than either resistance or aerobic exercise training alone [6,8,12], which is in line with the results of our pilot study [9]. Using a combined resistance and aerobic exercise training program has produced very good results, even for very sick patients, and it has demonstrated high long-term adherence rates [9]. Since there is evidence for better outcomes with higher frequency and longer duration of exercise [8,9,28], the targeted training frequency will be thrice-weekly for 12 months in this trial.

### 4.2. Clinical Endpoints

The choice of increased sit-to-stand repetitions in 60 s (STS60 test) as the primary endpoint is based on studies that indicate that it is a reliable parameter for the quantification of relevant changes in physical functioning in CKD patients [15]. The Six-Minute Walk Test (6MWT), the Timed-Up-and-Go (TUG) test and the Grip Strength Test (GST) have been shown to reflect the activities of daily living [6,8,29,30,31].

Improvement in quality of life is an important effect of exercise that is a high priority for patients treated with HD, which has been shown consistently in most trials of exercise interventions [32]. Assessing patients’ quality of life with the SF-36 instrument will therefore be an important secondary endpoint [33]. 

### 4.3. Safety

Exercise training during HD is generally considered safe if general precautions are followed [22,26]. This includes patients with a wide range of disabilities and comorbidities. Therefore, in DiaTT, we will apply only minimal restrictions on participation because of acute, uncontrolled conditions and adapt the exercise programs based on individual baseline exercise capacity, comorbidities and disabilities, such as amputations. The intensity, duration, repetitions and/or load of exercises will be gradually increased. In addition to regular dialysis staff, exercise therapists will be present during the exercise sessions. Adverse events that are possibly related to exercise (hypotensive episodes, leg cramping, disconnection/dislodging of catheters/dialysis needles) and hospitalizations/deaths (SAEs) will be recorded to analyze safety of intradialytic training compared to UC [11].

### 4.4. Health Economics 

Assessments of health economics are essential for the future implementation of an intradialytic exercise program into clinical routine. Cardiac rehabilitation after coronary artery bypass grafting in HD patients (analysis between 1998–2004; n = 4324 patients, 36 exercise sessions in 12 weeks, follow-up period 42 months) revealed a cost-effectiveness ratio of $13,887 per year of life saved at 42 months [34]. Currently, however, there is no evidence from large trials regarding the cost-effectiveness of intradialytic exercise interventions in HD patients. Therefore, by including a detailed cost analysis, DiaTT will substantially improve the scientific and economic evidence base in order to inform future implementation strategies, as has been demanded by experts in the field [5,10,11].

## 5. Conclusions

The syndrome of CKD, together with the treatment-related immobility associated to in-center HD, results in low cardiorespiratory fitness and very low physical activity levels for many patients on HD. Although small- and medium-sized trials have shown the benefits of exercise, evidence of the effectiveness of exercise programs that are suitable for a broad range of patients from large-scale trials is lacking. The DiaTT trial will be the largest cluster randomized, controlled trial assessing the efficacy of intradialytic training for CKD patients on HD. By testing an exercise program that is embedded in routine dialysis care without interfering with other care processes, it will have broad applicability in many settings of dialysis care. Thereby, the DiaTT trial will substantially contribute to the current understanding of exercise for CKD patients during HD; and it has the potential to change recommendations and guidelines and to improve physical and mental wellbeing for these patients. 

## Figures and Tables

**Table 1:** Visits and examinations during the DiaTT trial.

	Screening Visit 0	Baseline Visit 1	Visit 2	Visit 3	Visit 4	Visit 5
**Month (+/−2 weeks)**	−2	0	3	6	9	12
Informed consent	X					
Inclusion and exclusion criteria	X					
Randomization	X					
Physician’s confirmation	X					
Medical history	X	X				
Laboratory test (blood sampling)		X	X	X	X	X
Medication		X	X	X	X	X
Dialysis parameters		X	X	X	X	X
SAE/AE		X				
**Physical functioning tests**						
STS60		X	X	X	X	X
TUG		X	X	X	X	X
6MWT		X	X	X	X	X
GST		X	X	X	X	X
Ergometry ^#^				X		X
Weekly training sessions		X	X	X	X	X
**Health literacy questionnaire**						
HLS-EU-Q16		X				X
**Quality of Life/ Frailty questionnaire**						
SF-36		X	X	X		X
MPI		X	X	X		X
**Health economics (costs)**						
Medication		X				X
Hospitalisation		X				X
Mode of transportation		X				X
Therapeutic remedies		X				X
Medical assistance tools		X				X
Nursing		X				X
Sick leave from work-place		X				X

SAE: serious adverse event; AE: adverse event; STS60: Sit-To-Stand test; TUG: Timed-Up-and-Go test; 6MWT: Six-Minute Walk test; GST: Grip Strength Test; HLS-EU-Q16 health literacy survey; SF-36: short form 36; MPI: Multidimensional Prognostic Index; ^#^ only Intervention Group (IG).

## Registration Details

ClinicalTrials.gov NCT03885102 [registered 21 March 2019].
